# The Herbal Cocktail GSYJ Attenuated Airway Inflammatory Cell Infiltration in a Chronic Asthmatic Mouse Model

**DOI:** 10.1155/2021/6691475

**Published:** 2021-02-25

**Authors:** Chung-Jen Chiang, Shu-Lun Chang, Li-Jen Lin

**Affiliations:** ^1^Department of Medical Laboratory Science and Biotechnology, China Medical University, Taichung 40402, Taiwan; ^2^School of Chinese Medicine, College of Chinese Medicine, China Medical University, Taichung 40402, Taiwan

## Abstract

This study explored the potential therapeutic efficacy of GSYJ in attenuating asthma symptom severity and aimed to determine the immunomodulatory mechanism of GSYJ. A mouse model of chronic asthma induced by repeated *Dermatophagoides pteronyssinus* (Der p) challenge was established. In addition, 30 minutes before Der p challenge, the mice were orally administered GSYJ (1 g/kg). The mice were sacrificed to evaluate inflammatory cell infiltration, collagen deposition in the lung, total IgE in serum, and expression profiles of various cytokines in bronchoalveolar lavage fluid (BALF) and various genes in lung tissue. Furthermore, 30 minutes after the addition of GSYJ to RAW264.7 cell cultures, 100 ng/ml LPS was added to evaluate the effect of the drug on the LPS-induced expression of genes, proteins, and transcription factors. GSYJ may regulate transcription factors (cJUN/IRF3/NF-*κ*B) to decrease the expression of IL-1*β*, IL-6, RANTES, and iNOS in macrophages and affect the IL-12, IFN-*γ*, IL-5, and IL-6 levels in the BALF of mice to relieve asthma symptoms, such as inflammatory cell infiltration, hyperresponsiveness, and increased serum total IgE levels. Therefore, GSYJ has the potential to be developed into a drug treatment for chronic asthma.

## 1. Introduction

Asthma is characterized by chronic inflammation, airway hyperresponsiveness (AHR), and mucus secretion [1, 2]. Approximately 300 million people have asthma in the world, resulting in spending of billions of dollars in healthcare [3]. Approximately 53% of patients with asthma had a history of asthma attacks in the previous year, and 42% of patients had not attended school for more than one day or had not worked for a period of time due to worsening symptoms [4].

Currently, effective asthma prevention strategies or known cures are not available [5, 6]. When inhaled corticosteroid use is discontinued, the effects on asthma rapidly disappear [6]. In addition, inhaled corticosteroids affect linear growth, cataracts, bone density, bruises, and adrenal function [7]. Although inhaled corticosteroids often decrease asthma-related mortality, the prevalence of asthma is still increasing [8]. Clearly, new asthma drugs are needed to overcome the limitations of current treatments.

GSYJ is composed of four herbs: *Cinnamomum cassia* Presl., *Rehmannia glutinosa* Libosch., *Lycium chinense* Mill., and *Cuscuta australis* R. Br. *C. cassia* Presl., also known as Jou Kuei (Chinese name: 肉桂), is widely cultivated in China. The dry bark of *C. cassia* Presl. is an important food flavor and possesses antimicrobial, anti-inflammatory, antitumor, and antidiabetic properties [9]. *R. glutinosa* Libosch. is also known as Shan Zhu Yu (Chinese name: 熟地黃). It exerts anti-inflammatory, hypoglycemic, angiogenic, and hepatoprotective effects [10]. *C. australis* R. Br. is known as Yu Si Zi (Chinese name: 菟絲子), and its seeds have been shown to contain mainly kaempferol and astragalin. In addition, *C. australis* R. Br. has been shown to exert hepatoprotective and antioxidant effects on acetaminophen-induced hepatotoxicity in rats [11]. *C. australis* R. Br. has been used as a tonic to treat urinary complaints, such as frequent urination and involuntary ejaculation [12]. *L. chinense* Mill., also known as Gou Qi Zi (Chinese name: 枸杞子), possesses antioxidant activity. Gou Qi Zi exerts antiaging, immunomodulatory, antifatigue, and antitumor effects [13]. In the present study, we show that the oral administration of GSYJ reduces asthma symptoms.

Macrophages are located throughout human tissues and are responsible for degrading and processing foreign objects and dead cells. In addition, macrophages rapidly change their functions and recruit more macrophages in response to signals from the local microenvironment [14]. At the same time, macrophages are the most abundant white blood cells in the respiratory tract and are essential for regulating the immune response. Macrophages are the key to the development of allergic asthma. They promote inflammation and are accompanied by goblet cell hyperplasia, fibrosis and lung injury [15]. Macrophages in the lungs also secrete many factors that stimulate contraction of airway smooth muscle and degradation of ECM, leading to airway remodeling [15]. On the other hand, lung macrophages produce cytokines and chemokines to recruit Th2 cells, eosinophils, and basophils to the lungs, subsequently aggravating the severity of the disease [15]. Therefore, research designed to improve the function of macrophages will provide new strategies for the treatment of asthma.

## 2. Materials and Methods

### 2.1. Mice and Reagents

All animal experiments and healthcare comply with the regulations of the Institutional Animal Care and Use Committee of China Medical University (No. 2019-112). Six- to eight-week-old male mice (BALB/c) had no specific pathogens (National Laboratory Animal Center of the Republic of China). Ether was used to extract the Der p (Allergon, Sweden).

### 2.2. GSYJ Preparation

GSYJ was prepared from four common Chinese herbal medicines at a specific weight ratio (Table 1). Dr. Chin-Jen Wu, a Quality Assurance Manager from Kaiser Pharmaceutics Co., Ltd., identified the origin of the plant. The total amount of herbs used in this study was 360 g. All herbs were decocted twice with 2.52 L and 1.8 L of water for 1 hour. GSYJ was dissolved in deionized water and stored at −20°C.

### 2.3. Identification and Determination of the Contents of GSYJ Compounds

**Figure 1:**
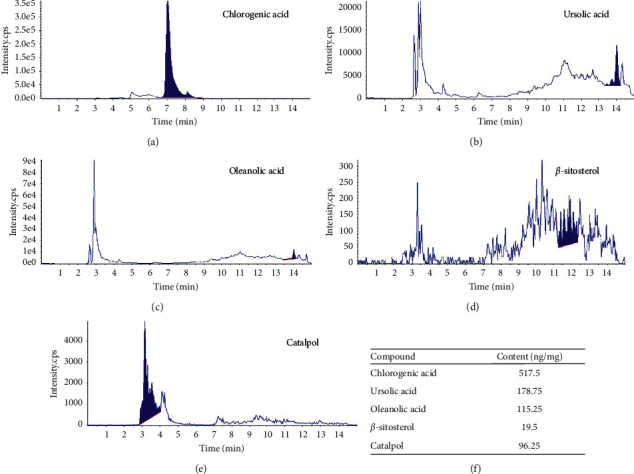
LC-MS analysis of the concentrations of the following compounds in GSYJ concentration of the compounds in GSYJ: (a) chlorogenic acid, (b) ursolic acid, (c) oleanolic acid, (d) *β*-sitosterol, and (e) catalpol (unit: ng/GSYJ ex. 1 mg) and (f) content of the compounds.

A 1 mg/ml standard (Sigma) solution was dissolved in methanol (HPLC grade, Merck). All standards were mixed, and methanol was used for serial dilution. All samples were stored at 4°C before use. The mixed standards were serially diluted to obtain different concentrations and then were used for plotting standard curves. Each sample solution was subjected to protein precipitation with 2x the volume of methanol and centrifuged at 15000xg for 10 minutes. Then, the supernatant was diluted 10x with methanol, vortex mixed, and passed through 0.22 *µ*m filter paper before injection. The optimized mass spectrometry parameters and HPLC conditions were similar to those of a previous study [16] (Figure 1).

### 2.4. Allergen Challenge and Airway Inflammation Assessment

As described in a previous study [16], mice were repeatedly challenged with Der p (1 mg/ml, 50 *μ*l) for a period of 6 weeks (seven times at 1-week intervals). The daily dose for a 70 kg clinical adult patient is 6 g of the Chinese medicinal formula, and the human dose converted for mice is 1 g/kg (the dosage for mice: 6 g of GSYJ/70 kg (human) x 12.7 = 1.089 g/kg micel the dose ratio from human to mice is 12.7) [16]. Mice in the GSYJ group were orally administered 1 g/kg GSYJ 30 minutes before the intratracheal administration of Der p. Mice in the PBS group were orally administered water and challenged with PBS. BALF and serum sample collection, cytospin preparation, and the method used to count total leukocytes were similar to those described in a previous study [16].

### 2.5. Measurement of Airway Hyperresponsiveness

The whole-body plethysmograph (Buxco Electronics, Inc., Troy, NY) has been used in accordance with the AHR manufacturer's protocol. Mice were exposed in different doses of nebulized methacholine (Sigma-Aldrich, St. Louis, MO) for 3 minutes, and the Penh value was measured.

### 2.6. ELISA

The IL-1*β*, IL-5, IL-6, and IL-12 levels were measured using Ready-SET-Go! ELISA (eBioscience, San Diego, CA), RANTES, and IFN-*γ* levels were measured using DuoSet! ELISA (R & D Systems, Abingdon, UK) and IgE levels were measured using an ELISA kit (BD Pharmingen) according to the manufacturers' protocols. Der p-specific antibodies were measured in a similar manner as IgE levels using 2 *μ*g/ml Der p antigen instead of the capture antibody and biotinylated rat anti-mouse IgG1 or IgG2a/2b mAb (2 *μ*g/ml; BD Pharmingen). After the completion of the color reaction, the result was scanned and recorded by using an ELISA recorder at OD_450_.

### 2.7. Collagen Analysis

One hundred milligrams of mouse lung tissue were homogenized in liquid nitrogen, and the homogenized lung tissue was extracted with 2 ml of HBS. Collagen in the supernatant was collected and quantified using a Sircol Collagen Assay kit (Biocolor, Belfast, UK).

### 2.8. Macrophage (RAW264.7) Culture and Gene and Protein Expression Analyses

RAW264.7 cells (Bioresource Collection and Research Center, Hsin-Chu, Taiwan) were cultured with a medium (high-glucose DMEM (Gibco, Grand Island, NY, USA), 10% FBS, and 1% P.S.) in an atmosphere containing 5% CO_2_ at 37°C. RAW264.7 cells were pretreated with various concentrations of GSYJ in the presence or absence of 100 ng/ml LPS. The cells were harvested at the indicated time points for quantitative PCR and western blotting. Cell supernatants were collected for ELISA and NO measurements.

### 2.9. Semiquantitative RT-PCR

The extraction of total RNA, reverse transcription of total RNA, and real-time PCR procedure were performed using methods similar to those described in a previous study [16]. *β*-Actin and GAPDH were used as controls for lung tissue and RAW264.7 cells, respectively. The gene-specific primers were as follows: *β*-actin, 5'-GGA AAT CGT GCG TGA CA-3', and 5'-CAC AGG ATT CCA TAC CCA AG-3'; IL-6, 5'-CTT CAC AAG TCG GAG GCT TA-3', and 5'-TTG GTA GCA TCC ATC ATT TCT TT-3'; IL-1*β*, 5'- CTG TCC TGT GTA ATG AAA GAC G -3', and 5'-TGC TCT GCT TGT GAG GT -3'; RANTES, 5'-AGA AGT GGG TTC AAG AAT ACA T -3', and 5'-GGA CCG AGT GGG AGT AG -3'; and inducible nitric oxide synthase (iNOS), 5'-GAG ACG GAT AGG CAG AGA TT -3', and 5'-GAG GAG CTG ATG GAG TAG -3'.

### 2.10. Western Blotting

The collected RAW264.7 cells (1.5 × 10^6^ cells/well) were lysed with lysis buffer (50 mM Tris (pH 8.0), 5 mM EDTA (pH 8.0), 150 mM NaCl, 0.1% SDS, 1% NP-40, and protease inhibitor cocktail. The concentration of protein was quantified using the Bradford analysis method (Bio-Rad). Proteins were mixed with SDS sample buffer (Invitrogen, Carlsbad, CA) and then separated on 9% SDS-PAGE gels. The SDS-PAGE gel, PVDF membrane, and filter paper were correctly stacked in the electrophoresis transfer tank, and the proteins were transferred at 130 V for 1 hour. The PVDF membrane was washed with PBST 3 times for 5 minutes, and a properly diluted primary antibody (Cell Signaling Technology, Boston, MA, USA) in PBST was incubated with the membrane at 37°C for 1 hour. The membrane was washed with PBST 3 times for 10 minutes. An HRP-conjugated secondary antibody was added and reacted for 1 hour at 37°C, and the membrane was washed with PBST 3 times for 10 minutes. Finally, protein was detected with an ECL kit.

### 2.11. NO Level

Fifty microliters of the cell supernatant or standard (0.1 M sodium nitrite) and 50 *µ*l of the solution (0.1% sulfanilamide in 5% phosphoric acid) were added to 96-well plates, and the mixture was reacted for 10 minutes in the dark. After 10 minutes, 50 *µ*l of a 0.1% N-1 naphthylethylenediamine dihydrochloride solution was added and reacted for 10 minutes in the dark. The OD was subsequently measured at 540 nm.

### 2.12. Immunofluorescence Staining

RAW264.7 cells were incubated with LPS and different concentrations of GSYJ for 60 min, fixed with 4% paraformaldehyde for 20 minutes, and then, permeabilized with 0.2% Triton-X for 20 minutes. The cells were incubated with the primary anti-NF-*κ*B/p65 antibody (Santa Cruz Biotechnology Inc. Dallas, TX, USA), Alexa Fluor 488-conjugated secondary antibody, and DAPI for 1 hour each at room temperature. Fluorescence was observed under a fluorescence microscope (original magnification, x40; Leica Microsystems, Wetzlar, Germany).

### 2.13. Statistical Analysis

The differences between each group were analyzed by Student's t-test. A *P* value <0.05 was considered significant.

## 3. Results

### 3.1. Effects of GSYJ on Inhibiting AHR and Airway Inflammation in a Mouse Model of Chronic Asthma

**Figure 2:**
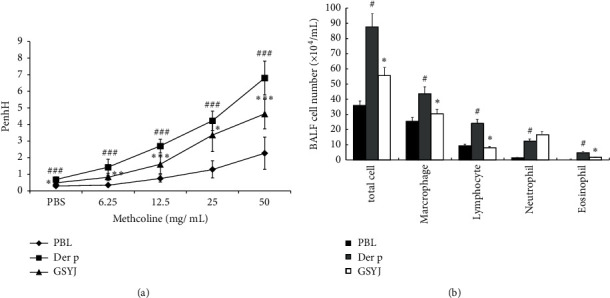
The effects of GSYJ on a mouse model of chronic asthma. (a) The anti-AHR effects of GSYJ. (b) The effects of GSYJ on inflammatory cell infiltration in the lung. Changes in the total cell number and cell population distributions in the BALF of mice sacrificed 72 hours after the final Der p challenge. BALB/c mice were challenged and assayed as described in the methods section. Values are presented as means ± SD of 6 mice. ^#^*P* < 0.05 and ^###^*P* < 0.001 compared with the PBS group; ^*∗*^*P* < 0.05, ^*∗∗*^*P* < 0.01, and ^*∗∗∗*^*P* < 0.001 for the comparison between the nontreated and GSYJ-treated groups.

Dyspnea is a characteristic symptom and one of the causes of death in patients with asthma. Therefore, new asthma drugs should relieve dyspnea. We tested the effects of GSYJ on AHR and inflammation in a repetitive Der p challenge model. We measured methacholine-induced Penh values as an indicator of AHR to bronchoconstriction in live mice. The Penh values of mice in the Der p group were higher than those in the PBS group. In the GSYJ group, the Penh value of the maximum dose of methacholine was significantly lower than that in the Der p group (Figure 2(a)). Moreover, macrophages were the main cells present in the BALF of mice in the PBS group, and no eosinophils were detected. Mice in the GSYJ group had significantly lower numbers of total cells, lymphocytes, macrophages, and eosinophils than mice in the Der p group (Figure 2(b)).

### 3.2. The Effects of GSYJ on Serum Total IgE, Der p-Specific IgG1, and Der p-Specific IgG2a/2b Antibodies

**Figure 3:**
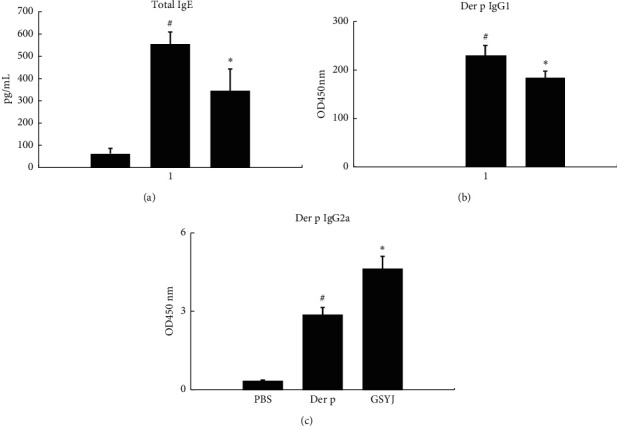
The effects of GSYJ on Der p-induced antibody production in the serum. The levels of total IgE, Der p-specific IgG1, and IgG2a/2b were measured using ELISA. Values are presented as means ± SD of 6 mice.

Patients with asthma present increased total serum IgE levels, which are a major symptom of asthma. We assayed the serum levels of total IgE, Der p-specific IgG1, and Der p-specific IgG2a/2b antibodies. Mice in the Der p group had significantly higher antibody levels than those in the PBS group. However, the serum levels of total IgE and Der p-specific IgG1 were significantly lower in the GSYJ group than in the Der p group. In addition, the level of Der p-specific IgG2a/2b antibodies was slightly higher in mice in the GSYJ group than in mice in the Der p group (Figure 3).

### 3.3. Immunoregulatory Effect of GSYJ on Der p-Induced Changes in the Cytokine Levels in BALF

**Figure 4:**
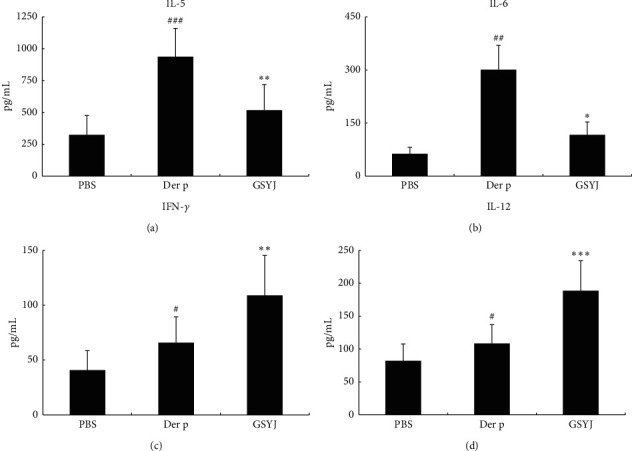
The effects of GSYJ on Der p-induced cytokine production in the BALF. IL-5, IL-6, IFN-*γ*, and IL-12 levels were measured using ELISA. Values are presented as means ± SD of 6 mice.

Many clinical trials have shown that TCM relieves asthma symptoms by regulating cytokine levels [17]. As shown in Figure 4, significantly lower levels of IL-5 and IL-6 were detected in mice from the GSYJ group, and the levels of IL-12 and IFN-*γ* were significantly higher than in mice in the Der p group.

### 3.4. Immunoregulatory Effect of GSYJ on Der p-Induced Gene Expression of Cytokine and Chemokine in Lung Tissues

**Figure 5:**
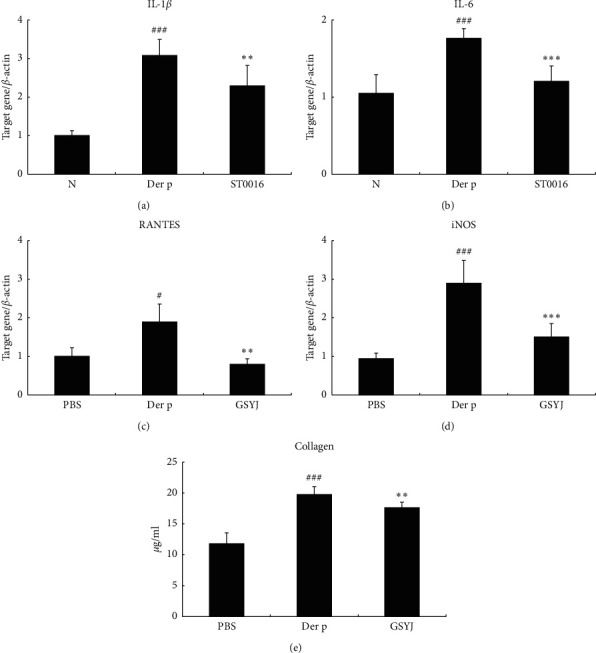
The effects of GSYJ on lung tissues from mice with Der p-induced chronic asthma. (a) GSYJ exerts suppressive effects by regulating the expression of IL-1*β*, IL-6, RANTES, and iNOS in lung tissue. (b) The effects of GSYJ on Der p-induced collagen deposition in mouse lung tissue. Collagen was present in the lung tissue. Values are presented as means ± SD of 6 mice.

After obtaining an understanding of the effects of GSYJ on asthma symptoms, we further studied the effects of GSYJ on lung gene expression profiles. Expression levels of the IL-1*β*, IL-6, and RANTES mRNAs were markedly higher in the Der p group than those in the PBS group. The GSYJ group mice showed significantly lower expression levels of the IL-1*β*, IL-6, and RANTES mRNAs than mice of the Der p group (Figure 5).

### 3.5. The Effects of GSYJ on Der p-Induced Collagen Deposition and iNOS Levels in the Lung Tissue

One characteristic feature of asthmatic airways is matrix deposition in subepithelial regions of the airway. We measure total collagen levels to evaluate the effect of GSYJ on matrix deposition within the lung tissue. The quantities of total collagen were markedly lower in the GSYJ group than in the Der p group (Figure 5). Based on this finding, the GSYJ treatment reduced collagen deposition in the allergic airway. In addition, iNOS levels were lower in mice in the GSYJ group than in mice in the Der p group.

### 3.6. Effects of GSYJ on LPS-Induced Changes in the mRNA and Protein Levels of Immune Response-Associated Genes in RAW264.7 Macrophages

Macrophages amplify the inflammatory response by recruiting inflammatory cells. We speculated that the GSYJ treatment reduces the total number of cells in the BALF (Figure 2(b)), which might be related to the immunoregulatory activity of GSYJ.

**Figure 6:**
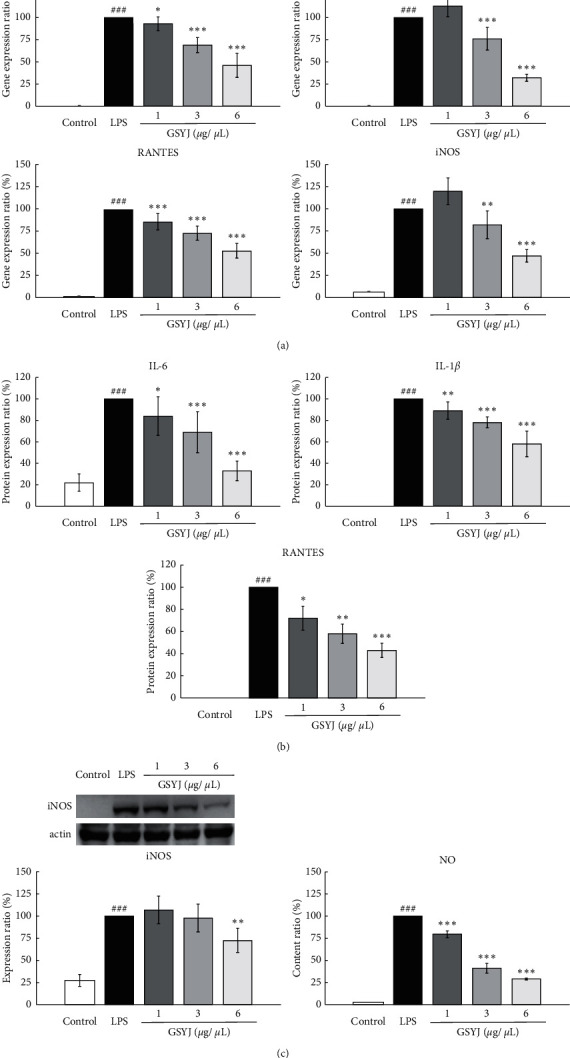
Effect of GSYJ on RAW264.7 macrophage cells stimulated with LPS. (a) Cells were incubated with GSYJ for 30 minutes and then stimulated with LPS for 18 hours. GSYJ exerts a dose-dependent inhibitory effect on IL-1*β*, IL-6, RANTES, and iNOS mRNA expression. (b) Cells were incubated with GSYJ for 30 minutes and then stimulated with LPS for 24 h. Levels of the IL-1*β*, IL-6, and RANTES proteins were measured using ELISA. (c) Cells were incubated with GSYJ for 30 minutes and then stimulated with LPS for 24 hours and 48 hours prior to iNOS and NO assays, respectively. These profiles are representative of profiles obtained from three independent experiments.

RAW264.7 cells were pretreated with GSYJ for 30 minutes. The subsequent analysis showed a significant, dose-dependent decrease in the expression of the IL-6, IL-1*β*, iNOS, and RANTES mRNAs and proteins after GSYJ treatment (relative to LPS treatment alone; Figure 6). In addition, lower NO levels were detected after the GSYJ treatment than after the LPS treatment (Figure 6(c)).

### 3.7. GSYJ Affected the Expression of Transcription Factors in RAW264.7 Macrophages

**Figure 7:**
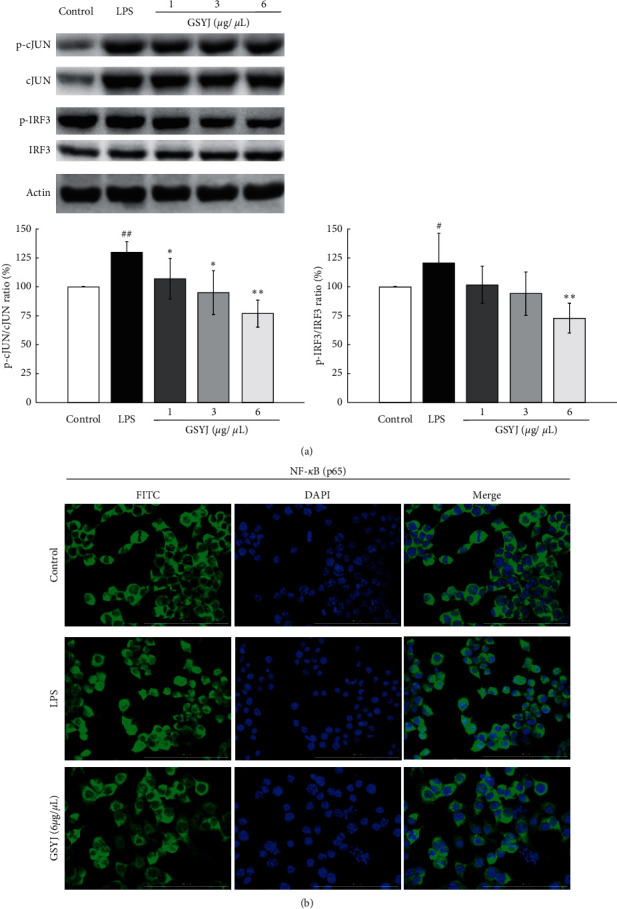
Effect of GSYJ on the expression of transcription factors in RAW264.7 macrophages stimulated with LPS. (a) GSYJ reduces the levels of p-c-Jun, c-Jun, p-IRF3, and IRF3. (b) GSYJ reduces the expression of NF-*κ*B in the nucleus. These profiles are representative of three independent experiments.

After LPS stimulation, macrophages activate three transcription factors, NF-*κ*B, AP-1 (MyD88-dependent pathways), and IRF3 (MyD88-independent pathways) to transmit signals [18]. GSYJ inhibits the expression of the IL-1*β*, IL-6, RANTES, and iNOS mRNAs in LPS-induced macrophages. Therefore, we examined whether GSYJ inhibited the expression of these genes by regulating the transcription factors JUN, IRF3, and p65. GSYJ significantly reduced the ratio of phosphorylated and unphosphorylated JUN and IRF3 (Figure 7(a)) and the expression of p65 in the nucleus (Figure 7(b)).

## 4. Discussion

Ethnopharmacological and traditional medicine uses more than 400 medicinal plants to inhibit and relieve the symptoms and progression of asthma and allergic disorders [19]. In the present study, we combined four medicinal materials with a new herbal cocktail for treating asthma. We used a mouse model of chronic asthma induced by repeated Der p challenge to confirm that GSYJ relieved AHR, cell infiltration in the lungs, and the amount of total IgE in serum (Figures 2 and 3). We used a macrophage cell line (RAW264.7) to explore the immunomodulatory mechanisms of GSYJ.

Many clinical trials have shown that TCM regulates cytokine production and reduces IgE levels. IL-5 is more specifically involved in the development and maturation, activation, survival, and migration of eosinophils [20]. Therefore, two main approaches have been developed: blocking circulating cytokines and interfering with IL-5 receptor alpha on eosinophils. Currently, three drugs are available to interfere with the action of IL-5: mepolizumab, benralizumab, and reslizumab [20]. The results of those drug clinical trials showed that patients with asthma had reduced exacerbation rates, an improved quality of life, decreased use of systemic steroids, and good safety [20, 21]. IL-6 is present at a higher concentration in the serum and BALF of patients with allergic asthma, and the concentration is negatively correlated with FEV1. Moreover, the presence of IL-6 in the lung airways is associated with impaired lung function [21]. Therefore, IL-6 may directly participate in the pathogenesis of asthma and increase the production of mucus in the lung airways [22]. Mouse experiments also confirmed that a lack of IL-6 reduces eosinophilic airway infiltration and allergen-induced AHR [23]. IL-12 mainly regulates the differentiation of TH1 cells and inhibits the expansion of TH2 cell clones. IFN-*γ* is involved in the inhibition of TH2 cells, such as eosinophil recruitment, bronchial hyperresponsiveness, and mucous goblet cell hyperplasia. Moreover, IL-12 and IFN-*γ* inhibit allergic inflammation and IgE synthesis in patients with asthma [24]. In the present study, the GSYJ treatment reduced the number of eosinophils in BALF, total IgE levels in serum, and AHR following Der p treatment (Figures 2 and 3). Therefore, we speculated that these results were attributed to the ability of GSYJ to inhibit the expression of the cytokines IL-6 and IL-5 and increase the expression of the cytokines IL-12 and IFN-*γ* (Figure 4).

Macrophages play a key role in the development of allergic asthma. Cytokines and chemokines produced by macrophages recruit eosinophils, basophils, and TH2 cells into the lungs. These changes will exacerbate the severity of the disease [15]. RANTES is constitutively expressed in the lungs and bronchoalveolar fluid of asthmatic patients. In addition, the expression of RANTES is related to the severity of the disease. RANTES mediates the migration and recruitment of eosinophils, neutrophils, and monocytes to the airways and triggers important events in the inflammatory response, such as antigen-specific IgE production and AHR. Moreover, preclinical studies suggest that strategies targeting RANTES and its receptors potentially represent a therapy for asthma [25]. IL-1*β* induces eosinophils to secrete the main basic protein (MBP), which further causes airway smooth muscle to contract and produce mucus. Furthermore, evidence from several animal asthma models has suggested that treatment with drugs that neutralize IL-1*β* reduces AHR, inflammatory cell infiltration, and the levels of TH2 cytokines [26]. Therefore, we speculated that the immunomodulatory effects of GSYJ were mediated by the inhibition of the expression of IL-1*β*, IL-6, and RANTES in the RAW264.7 cell line and lungs of mice (Figures 5–7).

Patients with asthma not only exhibit increased expression of iNOS in airway epithelial cells but also increased NO levels in exhaled air. In fact, NO is considered an inflammometer in asthma because its content in exhaled air (FE_NO_) is proportional to the ratio of bronchial wall inflammation, eosinophils, or induced sputum eosinophilia and AHR [27]. Furthermore, iNOS inhibitors exert beneficial anti-inflammatory effects on various acute and chronic animal inflammation models. Therefore, selective and more effective iNOS inhibitors and NO donors have been designed and developed as new therapies for patients with asthma [28]. In the present study, GSYJ inhibited iNOS expression and NO production in RAW264.7 cells and mouse lungs (Figures 5–7).

As a result of the wide application of medicinal plant species in alternative medicine, the phytochemical and pharmacological properties of these plants have been investigated. In our recent study, we found that DFSG contains multiple compounds and exerts multitarget effects, as it regulates the cytokines IL-12, IFN-*γ*, IL-5, and IL-13 through the coordinated action of its various compounds [29]. The isolated ingredients of medicinal plants include steroids, flavonoids, phenylpropanoids, and alkaloids, and compounds such as catalpol [30], *β*-sitosterol [31], chlorogenic acid [32], oleanolic acid [33], and ursolic acid [34] exert pharmacological antiasthma, immunomodulatory, and anti-inflammatory effects, as verified using in vitro and in vivo bioassays. Therefore, we identified twenty-two different chemical constituents in GSYJ (data not shown). One milligram of GSYJ contained 96.25 ng of catalpol, 19.5 ng of *β*-sitosterol, 517.5 ng of chlorogenic acid, 115.25 ng of oleanolic acid, and 178.75 ng of ursolic acid (Figure 1). Therefore, studies investigating the pharmacological properties of GSYJ compounds with anti-inflammatory and anti-AHR activities will enable us to better understand the potential mechanisms by which GSYJ ameliorates the symptoms of asthma.

## 5. Conclusions

In the present study, we documented immunomodulatory mechanisms of GSYJ that relieve asthma symptoms, including AHR, lung inflammatory cell infiltration, and increased serum total IgE levels by regulating the expression of IL-5, IL-6, IFN-*γ*, IL-12, IL-1*β*, RANTES, and iNOS. Moreover, the oral administration of a dose of 1 g/kg GSYJ in mice was based on the conversion the Chinese medicinal formula dose for patients with asthma for 1 day performed by a Chinese medicine practitioner. Based on these findings, GSYJ might be developed as a clinical drug for the treatment of asthma. Five ingredients were identified in GSYJ, and we hypothesize that GSYJ may contain many pharmacologically active compounds that exert synergistic effects on multiple targets to alleviate asthma symptoms.

## Figures and Tables

**Table 1:** GSYJ medicinal material combinations and doses.

TCM materia medica	Botanical family	Botanical nomenclature	Plant part	Amount (g)
1. Gou Qi Zi (枸杞子)	Solanaceae	*Lycium chinense* Mill.	Fruit	8
2. Shan Zhu Yu (熟地黃)	Scrophulariaceae	*Rehmannia glutinosa* Libosch.	Root	4
3. Yu Si Zi (菟絲子)	Convolvulaceae	*Cuscuta australis* R. Br.	Seed	4
4. Jou Kuei (肉桂)	Lauraceae	*Cinnamomum cassia* Presl.	Cortex	2
Total amounts	—	—	—	16

## Data Availability

All experimental data are within this manuscript.

## Abbreviations

AHR: Airway hyperresponsiveness
BALF: Bronchoalveolar lavage fluid
Der p: *Dermatophagoides pteronyssinus*

## References

[1] Manni M. L., Mandalapu S., McHugh K. J., Elloso M. M., Dudas P. L., Alcorn J. F. (2016). Molecular mechanisms of airway hyperresponsiveness in a murine model of steroid-resistant airway inflammation. *The Journal of Immunology*.

[2] Lyu Y., Chen X., Xia Q., Zhang S., Yao C. (2020). Network pharmacology-based study on the mechanism of pinellia ternata in asthma treatment. *Evidence-Based Complementary and Alternative Medicine*.

[3] Ali R., Ahmed N., Salman M., Daudpota S., Masroor M., Nasir M. (2020). Assessment of quality of life in bronchial asthma patients. *Cureus*.

[4] Centers for Disease Control and Prevention (CDC) (2011). Vital signs: asthma prevalence, disease characteristics, and self-management education: United States, 2001-2009. *Morbidity and Mortality Weekly Report*.

[5] Huang L., Guo J., Li W. (2019). Probiotics, prebiotics, and synbiotics for the treatment of asthma. *Medicine*.

[6] Guilbert T. W., Morgan W. J., Zeiger R. S. (2006). Long-term inhaled corticosteroids in preschool children at high risk for asthma. *New England Journal of Medicine*.

[7] Kelly H. W., Sternberg A. L., Lescher R. (2012). Effect of inhaled glucocorticoids in childhood on adult height. *New England Journal of Medicine*.

[8] WHO *Global Strategy for Asthma Management and Prevention*.

[9] Chou S.-T., Chang W.-L., Chang C.-T., Hsu S.-L., Lin Y.-C., Shih Y. (2013). Cinnamomum cassia essential oil inhibits *α*-MSH-induced melanin production and oxidative stress in murine B16 melanoma cells. *International Journal of Molecular Sciences*.

[10] Han K., Bose S., Kim Y.-M. (2015). Rehmannia glutinosa reduced waist circumferences of Korean obese women possibly through modulation of gut microbiota. *Food & Function*.

[11] Folarin R. O., Omirinde J. O., Bejide R., Isola T. O., Usende L. I., Basiru A. (2014). Comparative hepatoprotective activity of ethanolic extracts of Cuscuta australis against acetaminophen intoxication in wistar rats. *International Scholarly Research Notices*.

[12] Park J., Lee C. (2000). *The Encyclopedia of Medicinal Plants*.

[13] Moon H. W., Park J. W., Lee K. W. (2017). Administration of goji (Lycium chinense Mill.) extracts improves erectile function in old aged rat model. *The World Journal of Men’s Health*.

[14] Murray P. J., Wynn T. A. (2011). Protective and pathogenic functions of macrophage subsets. *Nature Reviews Immunology*.

[15] Shapouri-Moghaddam A., Mohammadian S., Vazini H. (2018). Macrophage plasticity, polarization, and function in health and disease. *Journal of Cellular Physiology*.

[16] Kao S.-T., Wang S.-D., Lin C.-C., Lin L.-J. (2018). Jin Gui Shen Qi Wan, a traditional Chinese medicine, alleviated allergic airway hypersensitivity and inflammatory cell infiltration in a chronic asthma mouse model. *Journal of Ethnopharmacology*.

[17] Chan H. H. L., Ng T. (2020). Traditional Chinese medicine (TCM) and allergic diseases. *Current Allergy and Asthma Reports*.

[18] Guo H., Zhang Y., Cheng B. C. (2018). An ethanolic extract of the aerial part of Siegesbeckia orientalis L. inhibits the production of inflammatory mediators regulated by AP-1, NF-*κ*B and IRF3 in LPS-stimulated RAW 264.7 cells. *BioScience Trends*.

[19] Mali R. G., Dhake A. S. (2011). A review on herbal antiasthmatics. *Oriental Pharmacy and Experimental Medicine*.

[20] Bagnasco D., Caminati M., Ferrando M. (2018). Anti-IL-5 and IL-5Ra: efficacy and safety of new therapeutic strategies in severe uncontrolled asthma. *BioMed Research International*.

[21] Hirano T., Matsunaga K. (2018). Late-onset asthma: current perspectives. *Journal of Asthma and Allergy*.

[22] Garth J., Barnes J., Krick S. (2018). Targeting cytokines as evolving treatment strategies in chronic inflammatory airway diseases. *International Journal of Molecular Sciences*.

[23] Lin Y.-L., Chen S.-H., Wang J.-Y. (2016). Critical role of IL-6 in dendritic cell-induced allergic inflammation of asthma. *Journal of Molecular Medicine*.

[24] Chung K. F. (2001). Anti-inflammatory cytokines in asthma and allergy: interleukin-10, interleukin-12, interferon-*γ*. *Mediators of Inflammation*.

[25] Marques R. E., Guabiraba R., Russo R. C., Teixeira M. M. (2013). Targeting CCL5 in inflammation. *Expert Opinion on Therapeutic Targets*.

[26] Liao Z., Xiao H.-t., Zhang Y. (2015). IL-1*β*: a key modulator in asthmatic airway smooth muscle hyper-reactivity. *Expert Review of Respiratory Medicine*.

[27] Batra J., Chatterjee R., Ghosh B. (2007). Inducible nitric oxide synthase (iNOS): role in asthma pathogenesis. *Indian Journal of Biochemistry & Biophysics*.

[28] Mulrennan S. A., Redington A. E. (2004). Nitric oxide synthase inhibition. *Treatments in Respiratory Medicine*.

[29] Lin L.-J., Huang H. Y. (2020). DFSG, a novel herbal cocktail with anti-asthma activity, suppressed MUC5AC in A549 cells and alleviated allergic airway hypersensitivity and inflammatory cell infiltration in a chronic asthma mouse model. *Biomedicine & Pharmacotherapy*.

[30] Li Y., Wang H., Yang X. (2019). Effects of catalpol on bronchial asthma and its relationship with cytokines. *Journal of Cellular Biochemistry*.

[31] Mahajan S. G., Mehta A. A. (2011). Suppression of ovalbumin-induced Th2-driven airway inflammation by *β*-sitosterol in a Guinea pig model of asthma. *European Journal of Pharmacology*.

[32] Kim H.-R., Lee D.-M., Lee S.-H. (2010). Chlorogenic acid suppresses pulmonary eosinophilia, IgE production, and Th2-type cytokine production in an ovalbumin-induced allergic asthma: activation of STAT-6 and JNK is inhibited by chlorogenic acid. *International Immunopharmacology*.

[33] Kim S.-H., Hong J.-H., Lee Y.-C. (2014). Oleanolic acid suppresses ovalbumin-induced airway inflammation and Th2-mediated allergic asthma by modulating the transcription factors T-bet, GATA-3, ROR*γ*t and Foxp3 in asthmatic mice. *International Immunopharmacology*.

[34] Kim S.-H., Hong J.-H., Lee Y.-C. (2013). Ursolic acid, a potential PPAR*γ* agonist, suppresses ovalbumin-induced airway inflammation and Penh by down-regulating IL-5, IL-13, and IL-17 in a mouse model of allergic asthma. *European Journal of Pharmacology*.

